# LIFE-COURSE AND CONTEXTUAL FACTORS OF ADVANCE CARE PLANNING AMONG OLDER ADULTS WITH LIMITED INCOME

**DOI:** 10.1093/geroni/igad104.3452

**Published:** 2023-12-21

**Authors:** Christine Kimpel, Jana Lauderdale, David Schlundt, Mary Dietrich, Amy Ratcliff, Cathy Maxwell

**Affiliations:** Vanderbilt University, Nashville, Tennessee, United States; Vanderbilt University, Nashville, Tennessee, United States; Vanderbilt University, Nashville, Tennessee, United States; Vanderbilt University, Nashville, Tennessee, United States; Tennessee Valley Healthcare System VA, Nashville, Tennessee, United States; Vanderbilt University, Nashville, Tennessee, United States

## Abstract

**Background:**

Cumulative disadvantage across the lifespan can lead to income, health, and advance care planning (ACP) disparities, resulting in increased caregiver decisional distress and reduced healthcare equity during times of cognitive incapacity. ACP is the process of patient and familial learning, communication, and documentation of life-sustaining care and quality of life preferences after personal reflection, but ACP rates remain lower among older adults with limited income. To better understand how life-course factors influence these low ACP rates, we aimed to explore participant perceptions of healthcare and ACP barriers and facilitators through different life stages.

**Methods:**

We sampled older adults (aged 50+) with limited income from six community-based sites (N=20). Qualitative descriptive design was utilized to perform semi-structured, in-person interviews.

**Results:**

Themes emerged from the inductive inclusion of in-vivo codes and the deductive application of the Cumulative Disadvantage Theory. Mean age: 64.8 years old (SD: 6.8); 11 participants identified as female (55.0%); 16 identified as Black/African American (80.0%). Four themes emerged: 1) structural, life-stage, 2) social stressors and resources, 3) individual stress responses and 4) ACP readiness. Participants’ perceptions denoted that proactive planning inequities among older adults with limited income result from the dynamic interplay of multi-dimensional stressors, protective factors, and personal stress responses. Implications: Identified themes serve as a conceptual basis for future intervention development that is responsive to traumatic life-course experiences and supportive of building ACP resilience (i.e., adapting to ACP stress and actively planning for times of cognitive incapacity), as well as policy and practice implications.

